# Outcomes of Paediatric Elbow Dislocations

**DOI:** 10.5704/MOJ.1603.008

**Published:** 2016-03

**Authors:** H Kaziz, N Naouar, W Osman, MLB Ayeche

**Affiliations:** Sahloul Sousse University Hospital, Sahloul Sousse, Tunisia

**Keywords:** Elbow, dislocation, children, injuries, outcome

## Abstract

**Introduction:** Elbow dislocations are uncommon in children. The treatment and outcomes remains controversial. Materials and methods: We report retrospectively the clinical and functional results of elbow dislocations in children treated in Sahloul University Hospital. Both isolated or pure dislocations and those associated with other injuries were evaluated separately.

**Results:** Forty-eight children were selected based on inclusion criteria. All were unilateral injuries. Pure dislocations were observed in 19 cases, out of which according to the Robert’s criteria, 13 children (68%) had excellent, three (15%) good, two (10%) fair, and one (7%)poor outcome. Out of the 29 elbow dislocations associated with other injuries, 3 (10%) had excellent, 4 (13%) good, 8 (27%) fair and 14 (50%) poor result. Reviewing the functional results, better range of motion was found in elbows with pure dislocation compared to those with associated injuries.

**Conclusion:** Prolonged follow-up and effective rehabilitation programs are required for good outcomes.

## Introduction

Paediatric traumatic elbow dislocation, is an uncommon injury^1^. It is estimated to occur between 3% to 6% of all paediatric elbow injuries^1–4^. Dislocation, isolated and with associated injuries are often seen between 10 and 15 years of age^2^. This high rate at the second decade of life is explained by the partial closing of the growth plates around the elbow joint^2,3^. Besides, physes which are still open lead to dislocations associated especially with fractures of the distal humeral extremity^4^. Isolated elbow dislocations are usually associated with disruption of collateral ligaments ^1,3,4^. The most commonly used classification based on the direction of displacement of the radio-ulnar unit from the distal humerus was adopted for our patients^5^. Posterior dislocation is the most common in the literature with a high rate of the posterolateral type at roughly 70% from all paediatric dislocations^5^. Fractures and entrapment of epitrochlea are the most reported associated injuries^5.6^. Fractures of lateral humeral condyle occur rarely^1, 3, 5, 6^. The aim of our study was to compare the outcome of isolated elbow dislocations with those with associated injuries.

## Materials and Methods

We reviewed retrospectively all cases of paediatric elbow dislocation admitted and treated in our department between January 2010 and December 2014. Parents of children were aware of this study and had consented. Inclusion criteria were age less than 15 years at time of dislocation, with anteroposterior and lateral radiographs of the injured elbow and documentation of functional evaluation available at the last follow up. Exclusion criteria were patients with age more than 15 years at time of injury, incomplete radiographic findings and absence of patient Does this mean patient did not turn up for last follow-up or incomplete functional evaluation at the last follow up. All complications especially neurovascular complications, were documented. Therapeutic options were revised and mentioned for all cases. Anterior–posterior and lateral elbow radiograph findings were re-analyzed from the first views at the time of injury to the final outcome. All elbows dislocations were reduced immediately under general anesthesia in the emergency operating room. All cases of isolated elbow dislocations only needed closed reduction. Stability of each elbow was evaluated after reduction. All isolated elbows dislocations were stable after closed reduction. Elbow stabilization with arm-forearm casts for three weeks were applied in elbows that were diagnosed to be stable without requiring additional fixation. In all other cases with associated injuries such as fracture entrapment of the medial epicondyle (epithrochlea), fractures of the lateral humeral condyle and fractures of radial head or olecranon, stabilization was performed simultaneously with reduction and treatment of these injuries. For these cases, intraoperative fluoroscopic views were needed to have an idea about displacement and options of surgical treatment. Demographics, mechanisms of injury, other associated injuries, neurological and vascular injuries and complications noted during treatment and follow up periods were recorded and entered in a computerized database. At final follow-up, all these elbows underwent anteroposterior and lateral view radiographs of the affected elbow in order to assess any incorrect axial alignment of the elbow joint, growth disturbances and calcifications around the elbow joint. Persistent or recurrent joint instabilities or permanent pain were documented. Roberts criteria were used to evaluate functional results [7] ( Excellent: no symptoms, no limitation of extension/flexion in the elbow movement; Good: mild symptoms, not more than 108° of loss of movement; Fair: moderate symptoms and a loss of movement between 108°–308°; and Poor: severe symptoms and a loss of more than 308° of movement). The aim of our study was to compare the results of pure dislocations to those with associated injuries. Statistical analysis was carried out using the Chi Square Test. Significance was assumed for p value less than 0.05. The minimum follow up was 9 months (range 9 months–5 years).

## Results

**Fig. 1:**
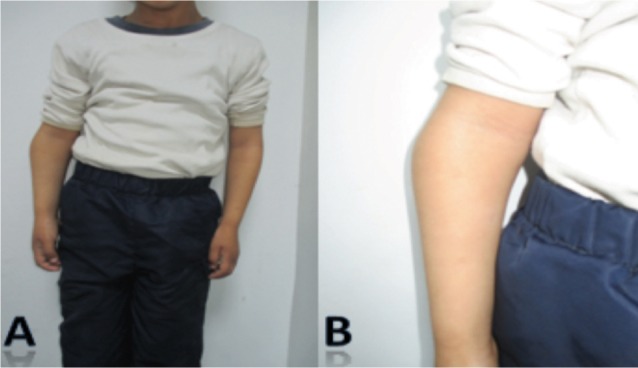
(A) Clinical presentation of the child in the emergency department with inability to move the sustained elbow. (B) the sustained elbow with swelling around the joint.

**Fig. 2:**
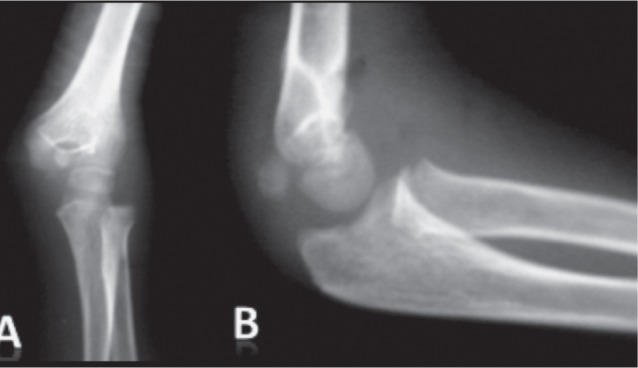
Anteroposterior (A) and lateral (B) views of the sustained elbow showing a lateral displacement of the radioulnar joint from the distal humerus with a fracture entrapment of the medial epicondyle.

**Fig. 3:**
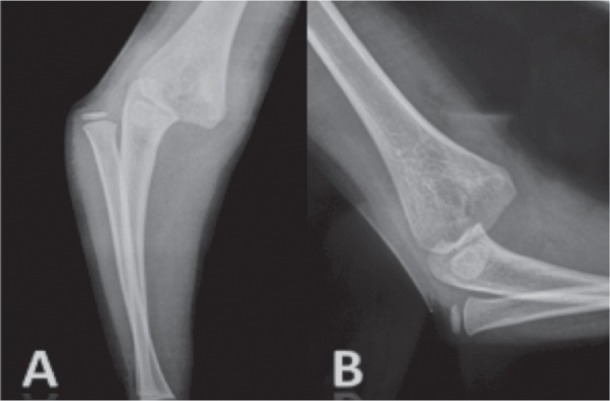
Anteroposterior (A) and lateral (B) views of the the sustained elbow revealing the posterolateral displacement of the radio-ulnar joint from the the distal humerus.

**Fig. 4:**
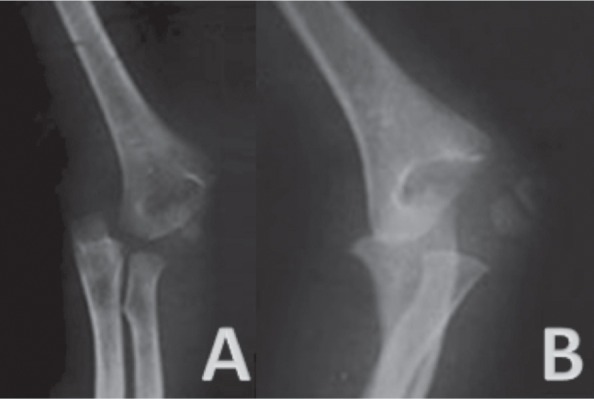
Anteroposterior (A) and lateral (B) views of the sustained elbow revealing the postero-medial dislocation of the elbow with a medial displacement of the proximal radio-ulnar joint medially from the distal humerus.

**Fig. 5:**
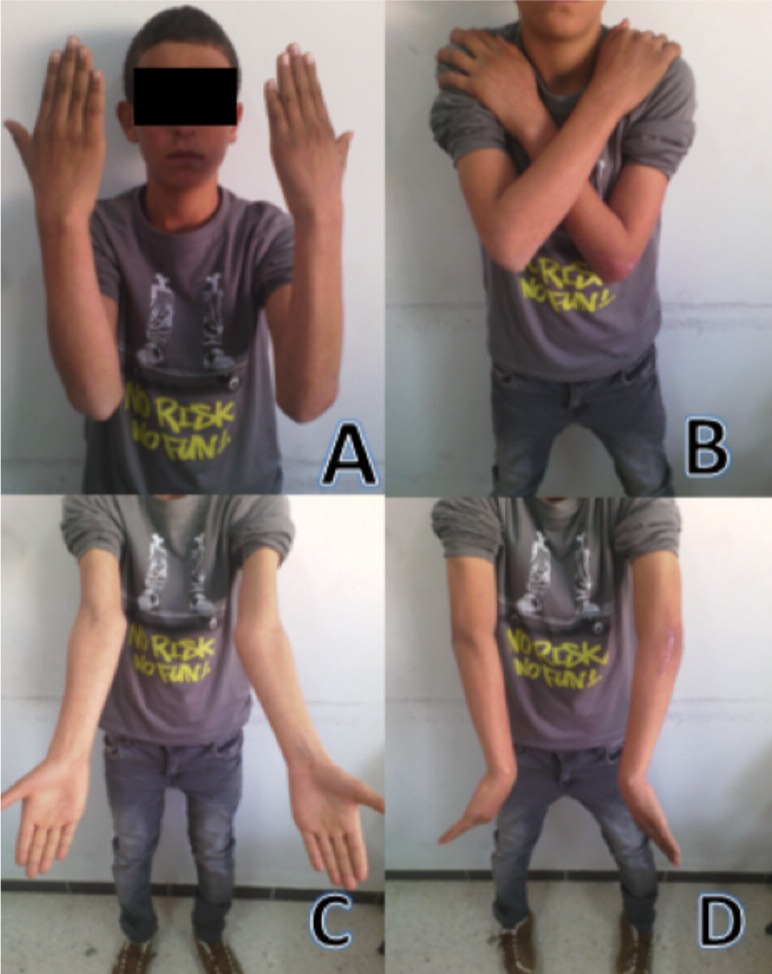
Evaluation of the functional outcome at final follow-up: A: flexion, B: flexion and pronation, C: supination, D: pronation.

**Fig. 6:**
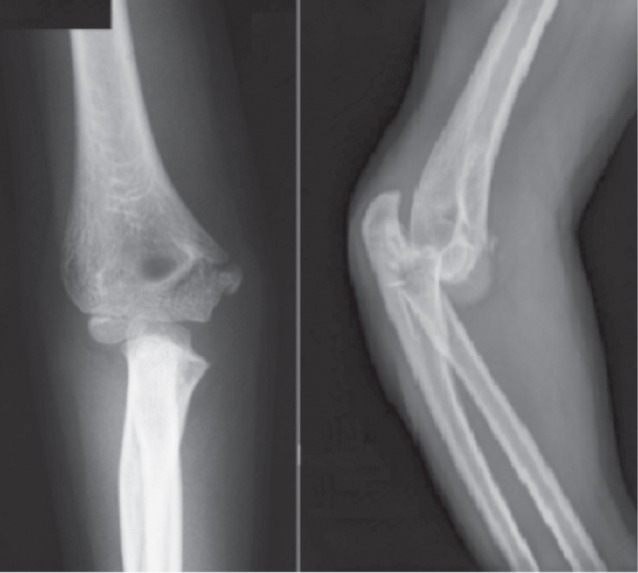
Anteroposterior and lateral radiographs views of the elbow showing a posterior dislocation of the elbow associated to fracture of the medial condyle.

**Fig. 7:**
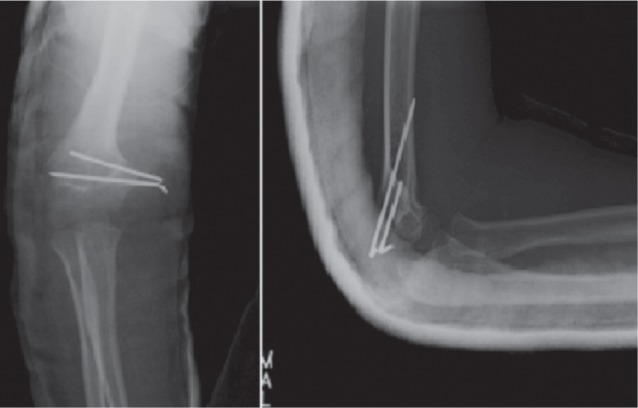
Post operative radiographs of the elbow after pinning of the medial condyle and immobilization of the joint with a plaster splint.

**Fig. 8:**
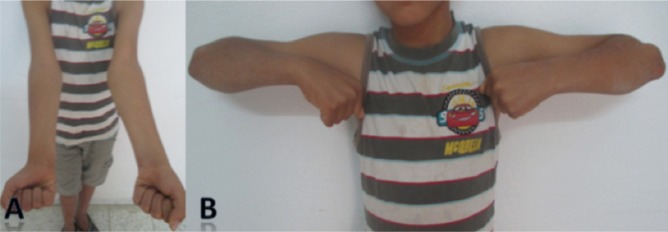
(A): Cubitus valgus complication with a lateral displacement of the forearm. (B) Absence of stiffness of the elbow inspite of the cubitus valgus.

**Fig. 9:**
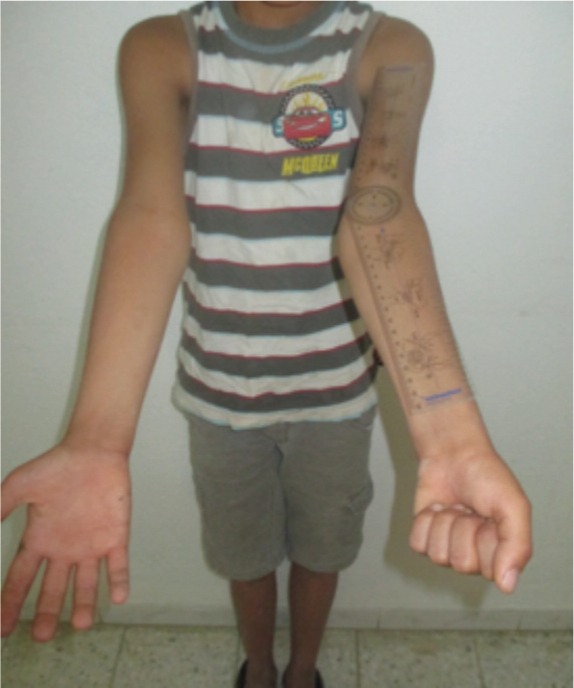
Carrying angle: The arm-forearm angle is measured clinically at each control for all patients. Elbow joint was in full extension, with the forearm supinated and hand/wrist in neutral position using a goniometer. The elbow was maintained at neutral, forearm in full supination and the wrist at neutral. An orthopaedic goniometer was placed with its hinge in the center of the cubital crease. The tips of the two axes of its arms were directed such that one was toward the lateral edge of the acromion (Easily palpable in children) and the other toward the midpoint of the radial and ulnar styloid. The angle was measured off the dial at the center of the goniometer, to the nearest degree (as that was the lowest count of the goniometer) this angle corresponded to the - acute angle between the axis of the arm and the axis of the fully supinated and extended forearm held neutral at the elbow.

We reviewed 61 paediatric elbow dislocations. However, as per our inclusion criteria, only 48 cases were included in the study. All were unilateral. Clinical presentation was the same for all patients with an elbow deformity and swelling around the joint (Figure 1).There were 31 boys and 17 girls with a mean age of 11.7 ( range 7–15) years. Dominant limb was involved in 32 cases. Twenty-nine children had right elbow dislocation and 19 left. The most common mechanism of injury was a direct fall on the outstretched hand. Only one dislocation was lateral (Figure 2), all others being posterior: of these 15 were pure posterior, 29 posterolateral (Figure 3) and three posteromedial (Figure 4). There were isolated or pure elbow dislocation in 19 cases without associated injuries. Mean age of these patients was 12.3 years (range 10–15 years). The pure dislocations were treated with closed reduction under general anesthesia with intraoperative fluoroscopic control. All of them were stable after reduction and were immobilized for a period of 3 weeks. At final follow-up, functional results (Figure 5) were documented according to Roberts criteria^7^. There were 13 children (68%) with excellent outcome, three (15%) - good, two (10%) fair, and one (7%) poor. The only observed complication was elbow stiffness documented in three cases that had fair and poor functional outcomes. No respect of recommendations after immobilization and no comply. Non-compliance with immobilisation and physiotherapy after plaster removal were the major causes of such results and also the low socioeconomic status of the parents. Twenty-nine cases had associated injuries. The mean age of those patients was 8.3 (range 6–12) years. Fracture of medial epicondyle was the most common associated injury, and was recorded in 13 cases. Entrapment of the fracture fragment in the joint was observed in four cases (Figure 2). Lateral condyle fracture was the second common associated injury in seven elbows. Four elbows had fracture of medial condyle which were treated with a simple pinning after open reduction (Figure 6, 7). There were three -others with avulsion fractures of coronoid and two with fracture of the radial head. No vascular complication had been reported. However, three ulnar palsy had been noted immediately after trauma and were diagnosed as neuropraxia and had recovered at an average of 45 days (range 27 – 60). No median nerve injury had been noted. Atrophy and contractures had developed on the patients’ forearm and hand leading to severe limitation of elbow movement in one patient and was classified as having poor result. All associated fractures had been covered in normal delay of consolidation. [Unable to understand this statement: delete? Eight elbows had been diagnosed with calcifications (myositis) around the joint. These calcifications were especially obvious in the medial part of soft tissue. Cubitus valgus deformity had been diagnosed in eight children (Figure 8). The mean carrying angle was 7.6° (Figure 9). This deformity was more evident in children having associated injuries especially of the medial condyle. Cubitus recurvatum was observed in two girls. According to Roberts criteria [7]: 3 (10%) had excellent, 4(13%) good, 8 (27%) fair and 14 (50%) poor result. Grouping all the patients together, 29 (60 %) of them had excellent or good, and 19 (40%) fair or poor result. Patients with closed reduction had a better outcome than those requiring open reduction. The functional results of both groups were compared statistically by Chi Square Test. There was a significant difference between two groups (p < 0.05).

## Discussion

Paediatric elbow dislocations are less common than fractures or epiphyseal injuries around the elbow joint^1^. Similar to the adults elbow dislocations, posterior variety is by far the most usual type of dislocation, explained as an effect of indirect forces transmitted to the elbow from a fall on the outstretched hand^7,8^. In our series, all dislocations were posterior with a dominance of posterolateral dislocations. Anterior and divergent dislocations are scarcely observed in paediatric elbow trauma^3, 5, 7, 9^. The mechanism explaining the occurrence of anterior elbow dislocation is a direct blow to the posterior aspect of the elbow, with avulsion of the insertion of the triceps from the olecranon^10^. Besides, convergent dislocation is the most frequent subgroup of the posterior dislocation variety^8^. In contrast, the divergent dislocation in which the ulna is reported to dislocate posteromedially and the radial head laterally to lie over the lateral condyle, is rarely seen in practice^8^. The suspected mechanism of injury is thought to be a combination of axial loading on the ulna and a strong pronation force causing disruption of the proximal radio-ulnar joint, the interosseous membrane and the annular ligament^5^. Paediatric elbow dislocations usually occur at the age of 13-14 years^10^. As with most paediatric injuries, boys are more commonly involved than girls^10^. Associated injuries and especially fractures occur in over half of posterior elbow dislocations ^10^. Carlioz and Abols had reported their earliest and largest series of elbow dislocation in children in which nearly 64% of dislocations were associated with other injuries around the elbow^11^. Fractures involving the medial epicondyle, radial head and neck, and coronoid process are the most reported types of associated injuries ^11^. However, fractures of the lateral epicondyle, lateral condyle, olecranon, capitellum, and trochlea may occur but less frequently^10^. Reviewing the literature, the most reported injury seems to be the fracture of the medial epicondyle^3,5,11^. The incidence of medial epicondylar fractures associated with elbow dislocation had been estimated to vary from 30% to 55% in many of the published articles concerning this entity ^3,5,9,10^. Several authors recommend a meticulous analysis of radiographic findings because the medial epicondyle is small and its displacement may be confused with the ossification centre especially of the trochlea or olecranon^4^. Treatment of such injury is controversial and no clear indications are established in literature. Both surgical and conservative treatments are advocated^5,9,10^. Surgical treatment is adopted by some physicians to prevent non-union and instability whilst others recommend surgery in the case of entrapment and in fractures with a displacement over 2 mm. According to Lieber, surgical treatment must always be indicated even in minimally displaced epicondyle fractures to ensure fracture union, prevent elbow instability and decrease the occurrence of valgus deformity^3^. In this same study, fixation of the medial epicondyle with screw is recommended to allow early mobilization and prevent elbow stiffness^3^. In our cases, we had K-wire insertion in eight of 13 medial epicondylar fractures Only three cases with an entrapment had progressed to fibrous union of the medial epicondyle with no elbow pain at final outcome. All our un-displaced fractures had recovered after the normal period. Treatment of other associated injuries such as the fractures of radial neck, lateral condyle, lateral epicondyle, coronoid, and olecranon are often managed non-operatively. Surgical treatment is indicated in case of displacement and complications^3,5,6^. Elbow dislocation in children is characterized by the occurrence of some complications. Some of them are immediately after injury such as vascular and neurological disruptions needing early exploration and others at follow up with growth disturbance abnormalities. Concerning vascular complications, it is rare that elbow dislocation lead to vascular disruption except those with open dislocation^5,11^, and rarely i in closed dislocations ^12^. It is reported that re-vascularisation procedures mostly would not be necessary because of adequate collateral circulation. If there is any concern of ischemia, excessive swelling at the elbow or a totally absent distal pulse, then surgical exploration is essential^11^. The ulnar nerve is more frequently affected in elbow dislocations^10^. These injuries are usually transient and resolve completely^5,10^. Three of our patients (6%) had ulnar nerve injury. Two patients’ symptoms resolved in four weeks but, the third child took eight weeks for full recovery. Some symptoms such as interosseous muscle atrophy, hypothenar atrophy, and mild contractures of the fingers occurred before total recovery. The median nerve palsy is infrequent but may occur and it is explained by its damage behind the medial epicondyle or within the joint after reduction^10, 13, 15^. Delay in diagnosis has been common^13^. Radial nerve injury was not observed in any of our patients. Later complications such as elbow stiffness, myositis ossificans, elbow instability, recurrent dislocations, radio-ulnar synostosis, cubitus valgus and cubitus recurvatum may occur during follow up. Immobilization of the elbow is compulsory after both open and closed reduction. Posterior splint is usually used. However, prolonged immobilization of the elbow can lead to stiffness and persistent limitation of the joint at follow up^3,10,16^. The duration of immobilization is controversial. Several authors found that immobilization should be limited to one week in case of pure dislocation whereas in case of associated injuries, stable fixation especially of the fractures should be performed and elbow mobilization as soon as possible^3^. Myositis ossificans is rare in children and when it occurs it is believed to be due to the disruption of the brachialis muscle during injury^10^. Some others factors had been reported in literature to have a direct effect in occurrence of myositis ossificans such as delay in treatment, long-term immobilization, and vigorous early active physiotherapy^3,5,10^. None of our patients was diagnosed to have developed myositis ossificans. A severe posterior elbow dislocation results in significant tearing of the anterior capsule. As a result, the patient may have some hyperextension (cubitus recurvatum) of the elbow ^17^. This is usually asymptomatic^10,17^. Recurrent dislocation is rare in children^18^. The occurrence of this complication is due to the failure of the capsule and ligamentous structures to be reattached after traumatic dislocation^5,18,19^. The rate of recurrence increases when treatment was delayed or incomplete^20^. Recurrence of dislocation was not seen in our series. Cubitus valgus is the most observed complication after elbow dislocation; it is more often seen in dislocations associated with other injuries leading to growth disturbance around the elbow^21^. Hinged elbow external fixators can be used for old dislocations after extensive soft tissue release^22^. However, the results of using hinged elbow external fixators for children with acute posterior elbow dislocations are unknown. Different rates have been reported for results of acute posterior elbow dislocation in children after treatment. The results reported in literature that were good or excellent ranged from 67% to 97%. Lieber *et al.*reported 100% excellent or good results for cases with pure dislocations, and 96% excellent or good results for cases with associated injuries. According to Lieber, this could be due to the low complication rates and the accurate diagnoses without treatment delay in all cases. In addition, no comorbid conditions such as neurovascular injuries occurred^3^. Rasool has reported 67% excellent or good, 30% fair, and 3% poor results^5^. Altuntas *et al.* have reported 7% fair, and only 3% poor results in their series^8^.

In our study, the number of patients with pure dislocation had better results than patients with associated fractures. Combined and associated injuries resulted in greater loss of movement than isolated fractures. Patients with closed reduction had better outcome than those requiring open reduction. Living in rural areas, with low socioeconomic levels, and avoiding routine visits were most commonly seen causing problems in the follow up period. In conclusion, we found that our success rate is lower than the rates reported in the literature. Prolonged follow up, good dialogue with families, and effective rehabilitation programs are required for good results in patients with posterior elbow dislocations. Otherwise, serious limitation in the range of elbow movement should be expected.

## Conclusion

Elbow dislocation in children is an uncommon injury. Some associated injuries may be seen and seem to lead to poor prognosis. Treatment of this injury depends on associated other injuries. Outcomes depend on the time of treatment and the surgical procedure.

## Conflict of Interest

No conflict of interest.
